# Beyond the hype - who uses cannabidiol for self-medication – and why: a cross-sectional study in Germany

**DOI:** 10.1186/s42238-025-00341-4

**Published:** 2025-10-15

**Authors:** Eva-Maria Krowartz, Carlotta Riemerschmid, Stefanie J. Klug, Luana F. Tanaka, Eva-Hoch

**Affiliations:** 1 Department of Psychiatry and Psychotherapy, University Hospital, Ludwig- Maximilians-University Munich, Munich, Germany; 2 IFT Institut für Therapieforschung (IFT), Centre for Mental Health and Addiction Research, Munich, Germany; 3 TUM School of Medicine and Health, Chair of Epidemiology, Technical University of Munich, Munich, Germany; 4 Paracelsus Medical University, Institute of Clinical Pharmacology, Nuremberg, Germany; 5 Department Clinical Psychology and Psychotherapy, Charlotte Fresenius University, Munich, Germany

**Keywords:** Cannabidiol, CBD, Self-Medication, Cannabis, Partial legalization

## Abstract

**Introduction:**

Cannabidiol (CBD) products are increasingly popular, marketed with health claims despite limited clinical evidence. This study investigates motives for CBD use in self-medication and explores sociodemographic characteristics and consumption patterns among regular users (at least monthly).

**Methods:**

Cross-sectional data were collected via an online survey (January 15–March 15, 2023) in Germany using convenience sampling. Associations between the motive of use (self-medication vs. recreational), sociodemographic characteristics, and consumption patterns were assessed using cross-tabulations, with Chi-square tests (*x²*) and Cramér’s V (*V*) for effect sizes. Self-medication predictors were analyzed using logistic regression, reporting adjusted odds ratios (aOR) and confidence intervals (CI).

**Results:**

A total of 730 participants participated in the study, with 702 (96.2%) complete cases included in the final analysis. The sample comprised 78.8% males with a mean age of 34.9 years (mean *SD*: 11.4). Among those using CBD primary for self-medication (37.9%, *n* = 266), the most prevalent motives were sleeping problems (52.3%), chronic pain (47.4%), depression (45.5%), and anxiety (44.4%). Motives for use were significantly associated with gender (*x²*=18.4, *V* = 0.162, *p* < .001), age (*x²*=42.3, *V* = 0.246, *p* < .001) and occupation (*x²*=51.8, *V* = 0.272, *p* < .001). In the adjusted logistic regression, older individuals (40–49 years, aOR: 1.97, CI: 1.05–3.70, *p* = .035; ≥50 years, aOR: 2.81, CI: 1.36–5.83, *p* = .005) and those unemployed or retired (aOR: 3.55, CI: 2.13–5.92, *p* < .001) were more likely to use CBD for self-medication. Higher chances were also observed in once-daily users (aOR: 2.52, CI:1.23–5.13, *p* = .011), those consuming CBD morning and evening (aOR: 3.05, CI: 1.42–6.56, *p* = .004), and individuals using it when needed (aOR: 2.72, CI: 1.72–4.29, *p* < .001). Smoking CBD mixed with tobacco (aOR: 0.37, CI: 0.22–0.62, *p* < .001) or pure CBD (aOR: 0.55, CI: 0.30–0.99, *p* = .046) was negatively associated with self-medication.

**Conclusion:**

This study identifies motives for CBD use in self-medication and examines associations with sociodemographic characteristics and consumption patterns. Clinical trials are needed to confirm efficacy, focusing on dose-response, administration and drug interactions. In the interest of users, the legal status—especially considering the recent partial legalization—should be clearly defined.

## Introduction

*Cannabis Sativa*has been utilized for medicinal, ritualistic, and recreational purposes for millennia (Russo 2007).The plant contains naturally occurring phytocannabinoids, which are key contributors to its pharmacological effects (Reekie et al. 2017). Cannabidiol (CBD), a phytocannabinoid synthesized via the secondary metabolism of*Cannabis sativa*(Pisanti et al. 2017), differs from tetrahydrocannabinol (THC), the most prominent cannabinoid, in that it does not activate cannabinoid-1-receptors. These receptors are primarily expressed in the brain and mediate the psychotropic effects commonly associated with cannabis consumption (Dingermann 2021).

In Germany, the lifetime prevalence of CBD use ranges from 4.3% (Alayli et al. 2022)to 11.4% (Geppert et al. 2020)in 2020, with CBD products gaining increasing popularity (Manthey 2023). CBD is primarily sold as dried flowers or as isolated CBD, which is processed into oil or incorporated into food and beverages. In Germany, the legal status of CBD products is complex und constantly changing (Kirsch 2023). Initially, the classification of CBD as a narcotic was evaluated. CBD dried flowers were considered narcotic substances due to their residual THC content and the possibility of misuse. Dried plant material often retains traces of THC above the legal threshold of 0.2% THC (Wissenschaftliche 2020). Courts contended that the potential for intoxication exists, thereby making the sale illegal, despite the economical impracticability due to the large amounts of dried flowers needed (Kirsch 2023). Following the partial legalization of recreational cannabis in April 2024, the legal status of CBD dried flowers in Germany has been redefined. The new legislation permits the sale of CBD dried flowers, provided they are derived from approved industrial hemp strains and do not exceed the newly defined THC limit of 0.3% (Beckmann 2024).

Other CBD-containing products, such as high-dose CBD oils with isolated CBD that may have pharmacological effects or are intended for disease treatment, are classified as medicinal products within the European Union (EU). These products must receive authorization in accordance with EU pharmaceutical regulations (European 2001). The therapeutic potential and potential adverse health effects of CBD have been widely debated, preclinical studies suggest that CBD interacts with various molecular targets, including receptors, enzymes, and ion channels, indicating potential therapeutic applications (Ibeas Bih et al. 2015). Nevertheless, large-scale randomized-controlled trials (RCTs) in humans remain limited (Britch et al. 2021; Ibeas Bih et al. 2015), and to date, the only approved medication containing the active ingredient CBD is*Epidyolex*, which received approval in September 2019 for the treatment of treatment-resistant epilepsy in Dravet syndrome, Lennox-Gastaut syndrome and tuberous sclerosis complex (Devinsky et al. 2017, 2016; GW 2021; Thiele et al. 2021, 2018).

In contrast, low-dose CBD products, such as food products, that are not intended for medicinal purposes are not regulated as pharmaceuticals (Lachenmeier 2019). Given that CBD alone does not exhibit psychoactive effects and is not considered an addictive substance, the European Commission concluded on December 3, 2020, that CBD should be classified as a novel food under*Regulation (EU) No. 2015/2283*(Lachenmeier 2020). Novel foods must undergo authorization by the European Food Safety Authority (EFSA). In 2022, the EFSA published a statement concluding that the safety of CBD as a novel food could not be determined due to existing uncertainties and data gaps (Nutrition et al. 2022). The Federal Office of Consumer Protection and Food Safety therefore concludes that CBD in food products, including dietary supplements, is currently not authorized for sale (6undefined ). Although considerable uncertainties remain regarding the regulatory status of CBD, Germany is expected to experience a significant increase in revenue within the CBD products market, with projections indicating a rise of 0.8% to approximately €395,41 million by 2025. When compared globally, Germany ranked second in 2024, following the United States (U.S), in terms of revenue generated from CBD retail sales (Statista 2024).

Due to the constantly changing and unclear legal status, unregulated over-the-counter CBD products are available in retail stores and online (McGregor et al. 2020), often being marketed illegally with unverified health claims (Engeli et al. 2025). Currently, no prevention campaigns specifically target misinformation about CBD products. While German consumer associations provide guidance (Verbraucherzentrale. 2025, 2023; Verbraucherzentrale 2024, 2019), manufacturers’ commercial interests often conflict with users’ interests. The ambiguous and evolving classification of CBD creates loopholes that producers repeatedly exploit to market their products with unverified claims (Melchert et al. 2024). Advertising, predominantly distributed via social media, primarily promotes CBD for pain, anxiety disorders, sleep disorders, and stress (Soleymanpour et al. 2021). Those advertised health claims are also reflected in users’ motivations for use. Self-medication has been reported for a variety of medical and psychological conditions. Cross-sectional studies in France (Fortin et al. 2021), the United Kingdom (UK) (Moltke and Hindocha 2021), the U.S (Corroon and Phillips 2018)., Germany (Geppert et al. 2020)and Poland (Binkowska et al. 2024)indicate that CBD is frequently used as a self-administered treatment for conditions such as stress, sleep disorders, pain, and mental health issues, including depression and anxiety (Binkowska et al. 2024; Corroon and Phillips 2018; Fortin et al. 2021; Geppert et al. 2020; Moltke and Hindocha 2021). Although CBD is widely available and frequently marketed for its health benefits, its use is not without potential risks. While large-scale human safety data remain limited, emerging evidence suggests a potential association between CBD use and increased rates of adverse events, including hepatotoxicity (Moore et al. 2024).

Given the potential risks associated with unregulated CBD products, the increasing demand, and recent regulatory changes following partial legalization, this study provides an exploratory analysis of regular German CBD users. Previous cross-sectional studies provide only limited empirical evidence on the sociodemographic characteristics and consumption patterns of regular users who primarily utilize CBD for self-medication. This study investigates the motives for CBD self-medication in a sample of regular users and examines sociodemographic factors and consumption patterns associated with self-medication.

## Methods

### Study design

The online survey was conducted from January 15 to March 15, 2023, using LimeSurvey (LimeSurvey GmbH) and employed convenience sampling to recruit regular CBD users in Germany. At the beginning of the questionnaire, participants provided informed consent for the use of their data for the specified research purposes. No personal data were collected, and participation was voluntary without the provision of incentives. The study sample comprised adult, self-selected CBD users in Germany who accessed the survey via a direct link or QR code. The inclusion criteria encompassed regular CBD consumption (at least monthly), residency in Germany, proficiency in German, and internet access to complete the survey. Regular CBD consumption and residency were verified using filter questions. Participants who did not meet the inclusion criteria received a notification of their ineligibility, and the questionnaire was subsequently terminated with a closing message. Recruitment was facilitated by CBD retailers and the German Hemp Association (DHV), who distributed the survey through platforms, such as YouTube, Instagram, and email newsletters. CBD retailers were identified via Google and were contacted to request their assistance in disseminating the survey through their channels. A search identified 84 retailers that were initially contacted by email. The overall response rate among CBD retailers was 57.1%, with 28 retailers (33.3%) in total supporting the recruitment process.

### Measurement

The questionnaire was based on that of Moltke et al. (Moltke and Hindocha 2021), translated into German, and adapted to the German population where applicable. It consisted of 23 questions and an additional open-ended item for further remarks. The questions were organized into three main domains: socioeconomic characteristics, motives for use, and patterns, and self-perceived effectiveness, safety, and acquisition. Methodological feedback was provided by the IFT Institut für Therapieforschung, Center for Mental Health & Addiction Research, while content-related insights were obtained from CBD retailers.

### Outcome

The main outcome was primary use motive, dichotomized into recreational use and self-medication. Participants selected either “*For medical reasons (to treat at least one illness or symptom)*” (self-medication) or *“For general health and well-being”* (recreational use). Based on their response, the subsequent question presented a predefined list of either recreational or self-medication motives, with multiple responses allowed. Self-perceived effectiveness was assessed among participants who reported self-medication as their primary use motive. Sociodemographic characteristics assessed included gender, age, education, and occupation, with single responses required. Regarding consumption patterns, participants were asked to report the frequency, route of administration, time of consumption, duration of use, and the amount consumed per day. For each item, predefined response categories were provided, with participants instructed to select one single category that most accurately reflected their behavior.

### Statistical analysis

Although vocational qualification was assessed, it was excluded from further analysis due to high multicollinearity with education and occupation, a factor crucial for regression modeling (Kim 2019). To ensure an adequate number of observations per category, particularly for regression modeling, some categories were collapsed. Participants who reported a non-binary gender were recoded as missing due to a low number of observations in this category. Education was recoded into three categories: ‘high school diploma or equivalent qualification’, ‘upper secondary education’, and ‘lower secondary education or below’. Occupation was recoded into the following categories: ‘employed full-time’, ‘employed part-time’, ‘pupil/student/intern or marginally employed’, and ‘not currently employed or retired’. Regarding the preferred route of administration, categories such as ‘as a drink’, ‘as an oral spray’, ‘as food’, ‘in a pipe’, ‘in a hookah’, ‘capsules or pills’, and ‘topically, on the skin’ were merged into the category ‘other’.

Cross-tabulations were performed to examine the association between motive of use, sociodemographic characteristics, and consumption patterns. Chi-squared (*x²*) tests assessed relationships, with Cramér’s V (*V*) used to measure effect size. *V*was applied to determine the strength of association between two nominally scaled variables, with values between 0.1 and 0.3 indicating a weak association, 0.4 to 0.5 indicating a moderate association, and values greater than 0.5 indicating a strong association (Cohen 1988). Binary logistic regressions were performed to assess associations between sociodemographic characteristics, consumption patterns, and the likelihood of reporting self-medication as the primary motive for CBD use (reference: recreational use), while controlling for other factors. To quantify the strength of associations and assess statistical significance, adjusted odds ratios (aORs) with 95% confidence intervals (CI) are reported. Variables that showed a significant association with motive of use, as identified by*V* and corresponding p-values (*p*), were included in the model. These predictors included gender, age, occupation, frequency and time of consumption, and route of administration. Overall model significance was tested using a *x²* statistic, which compares the null model (without predictors) to the model with predictors. The *x²*statistic is calculated by evaluating the difference between the observed and expected outcomes for each participant (Harris 2021).

## Results

In total, 730 individuals aged 18 to 80 fully completed the survey and received a completion message, after which the survey was terminated; these participants were considered valid cases. Participants with missing values in any of the variables under investigation were excluded, resulting in a final analytical sample of *n* = 702. Data analysis was performed using Stata (version 15.1). The sample consisted of 78.8% male users, with a mean age of 34.9 years (*range*: 18–80 years, mean *standard deviation*: 11.4). A detailed overview of sociodemographic characteristics and consumption patterns for the whole sample is provided in Table 1.

**Table 1:** Sociodemographic characteristics and consumption patterns in a self-selected sample of regular German CBD users (*n* = 702)

	Total sample (*n* = 702)	
	n	%
Gender		
Male	553	78.8
Female	149	21.2
Age groups (years)		
18–24	129	18.4
25–29	150	21.4
30–39	221	31.5
40–49	124	17.7
≥ 50	78	11.1
Education		
High school diploma	355	50.6
Upper secondary education	245	34.9
Lower secondary education or below	102	14.5
Occupation		
Employed full-time	412	58.7
Employed part-time	88	12.5
Pupil/student/intern or marginally employed	91	13.0
Not currently employed or retired	111	15.8
Frequency of use		
At least monthly	83	11.8
At least weekly	82	11.7
Several times per week	175	24.9
Once a day	133	19.0
Several times per day	229	32.6
Route of administration		
Sublingually	159	22.7
Smoking (with tobacco)	216	30.8
Smoking (pure)	121	17.2
Vaping	124	17.7
Other	82	11.7
Daytime of consumption		
In the evening	310	44.2
In the morning	21	3.0
Morning and evening	52	7.4
Multiple times per day	144	20.5
When needed	175	24.9
Consumption duration (years)		
< 1	95	13.5
1–2	190	27.1
2–5	297	42.3
> 5	120	17.1
Amount consumed per day (mg)		
< 100	197	28.1
100–300	142	20.2
> 300	91	13.0
Do not know	272	38.8

Around one third of the sample (37.9%, *n* = 266) reported using CBD primary for self-medication. A weak association between motive of use and sociodemographic factors, as indicated by V, was found for gender, age group, and occupation, while no significant association was observed for education. Male gender was associated with recreational CBD use, while female users were more likely to use CBD for self-medication. (*x²*=18.4, *V* = 0.162, *p* <.001). Additionally, a weak association was found between age group and motive of use (*x²*=42.3, *V* = 0.246, *p* <.001), with increasing age being associated with self-medication. Furthermore, motive of use was significantly associated with occupation (*x²*=51.8, *V* = 0.272, *p* <.001). A higher proportion of full-time employed participants were found among recreational users (65.4% vs. 47.7%), while individuals not currently employed or retired reported using CBD primarily for self-medication more frequently (28.2% vs. 8.3%). No significant association was observed between education and motive of use (*x²*=5.7, *V* = 0.090, *p* =.058). Regarding consumption patterns, V indicated significant associations between motive of use and specific consumption patterns. Motive of use was associated with frequency of use (*x²*=28.5, *V* = 0.202, *p* <.001), route of administration (*x²*=51.7, *V* = 0.271, *p* <.001), and the daytime of consumption (*x²*=45.5, *V* = 0.256, *p* <.001), all of which indicated weak effect sizes. In contrast, no significant associations were found between motive of use and consumption duration (*x²*=5.4, *V* = 0.088, *p* =.147), or the amount consumed per day (*x²*=3.6, *V* = 0.071, *p* =.311).

Sociodemographic characteristics and consumption patterns, stratified by primary consumption motive, are presented in Table 2, along with *V* and *p* to assess the magnitude and significance of differences.

**Table 2:** Sociodemographic characteristics and consumption patterns of regular cannabidiol users in Germany by primary use motive

	Self-medication (*n* = 266)		Recreational use (*n* = 436)		V ^1)^	*p*
	n	%	n	%		
Gender					0.162	< 0.001*
Male	187	70.3	366	83.9		
Female	79	29.7	70	16.1		
Age Groups (years)					0.246	< 0.001*
18–24	29	10.9	100	22.9		
25–29	48	18.1	102	23.4		
30–39	81	30.5	140	32.1		
40–49	58	21.8	66	15.1		
≥ 50	50	18.8	28	6.4		
Education					0.090	0.058
High school diploma	122	45.9	233	53.4		
Upper secondary education	96	36.1	149	34.2		
Lower secondary education or below	48	18.1	54	12.4		
Occupation					0.272	< 0.001*
Employed full-time	127	47.7	285	65.4		
Employed part-time	35	13.2	53	12.2		
Pupil/student/intern or marginally employed	29	10.9	62	14.2		
Not currently employed or retired	75	28.2	36	8.3		
Frequency of use					0.202	< 0.001*
At least monthly	21	7.9	62	14.2		
At least weekly	23	8.7	59	13.5		
Several times per week	51	19.2	124	28.4		
Once a day	60	22.6	73	16.7		
Several times per day	111	41.7	118	27.1		
Route of administration					0.271	< 0.001*
Sublingually	83	31.2	76	17.4		
Smoking (with tobacco)	52	19.6	164	37.6		
Smoking (pure)	30	11.3	91	20.9		
Vaping	58	21.8	66	15.1		
Other	43	16.2	39	8.9		
Daytime of consumption					0.256	< 0.001*
In the evening	78	29.3	232	53.2		
In the morning	12	4.5	9	2.1		
Morning and evening	32	12.0	20	4.6		
Multiple times per day	60	22.6	84	19.3		
When needed	84	31.6	91	20.9		
Consumption duration (years)					0.088	0.147
< 1	35	13.2	60	13.8		
1–2	60	22.6	130	29.8		
2–5	119	44.7	178	40.8		
> 5	52	19.6	68	15.6		
Amount consumed per day (mg)					0.071	0.311
< 100	83	31.2	114	26.2		
100–300	56	21.1	86	19.7		
> 300	35	13.2	56	12.8		
Do not know	92	34.6	180	41.3		

* Significant on a threshold of *α* < 0.05

^1)^ Cramér’s *V*: Strength of association between categorical variables (0 = no association, 1 = strong association)

**Fig. 1:**
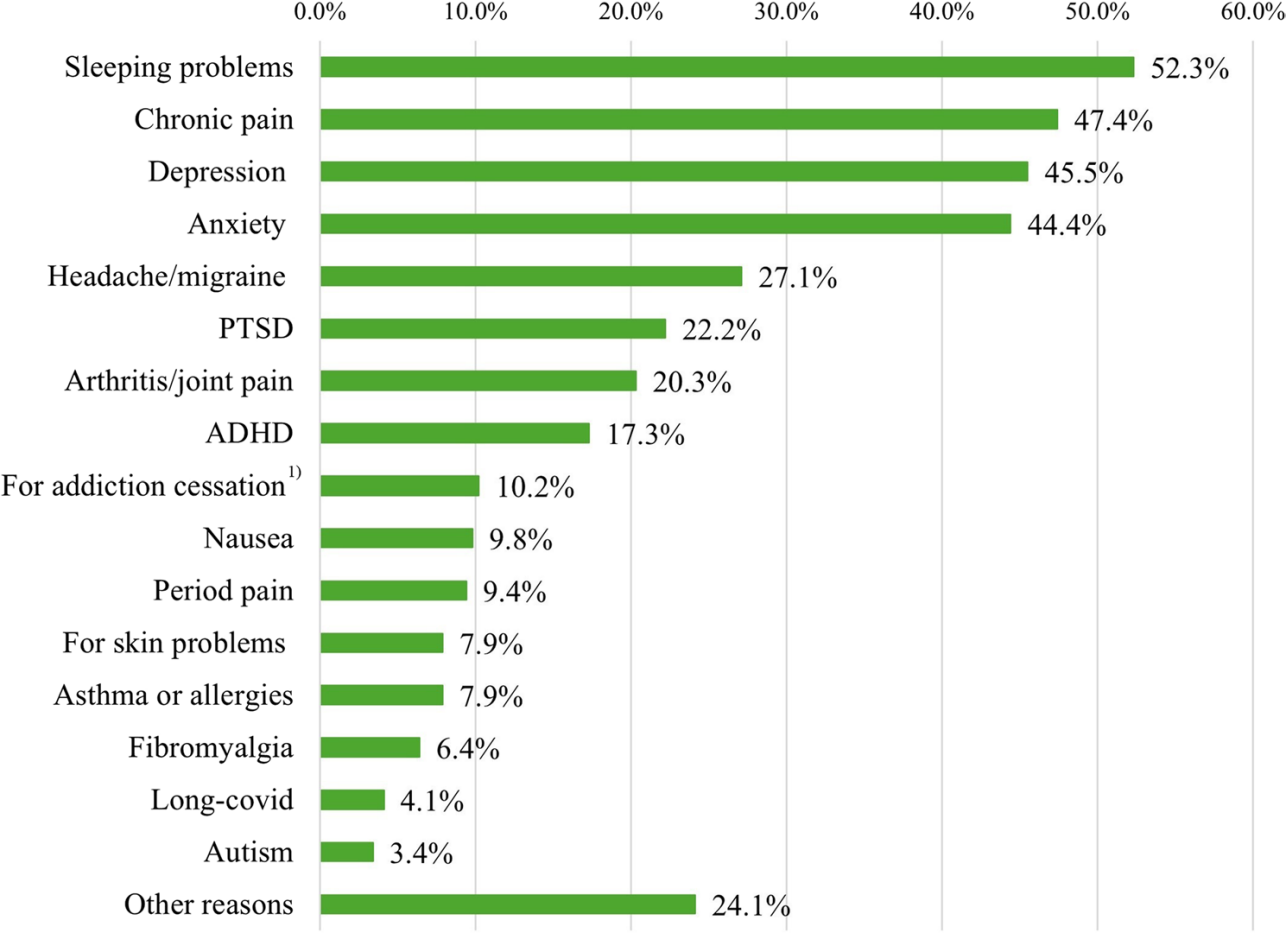
Reasons for self-medication among self-selected regular CBD users in Germany (multiple responses allowed) (*n* = 266). ^**1)**^ Substance not specified. ***Note:*** ADHD, attention deficit disorder; CBD, cannabidiol; COPD, chronic obstructive pulmonary disease; PTST, post-traumatic-stress disorder

Among individuals that reported using CBD primary for self-medication, users treated on average 3 to 4 different medical conditions with CBD (mean: 3,7). More than half of the primary medical users reported that they treated insomnia or other sleeping problems (52.3%), chronic pain (47.4%), depression (45.5%), or anxiety (44.4%) with CBD. The most common indications for primary medical CBD use are displayed in (Fig. 1).

**Fig. 2:**
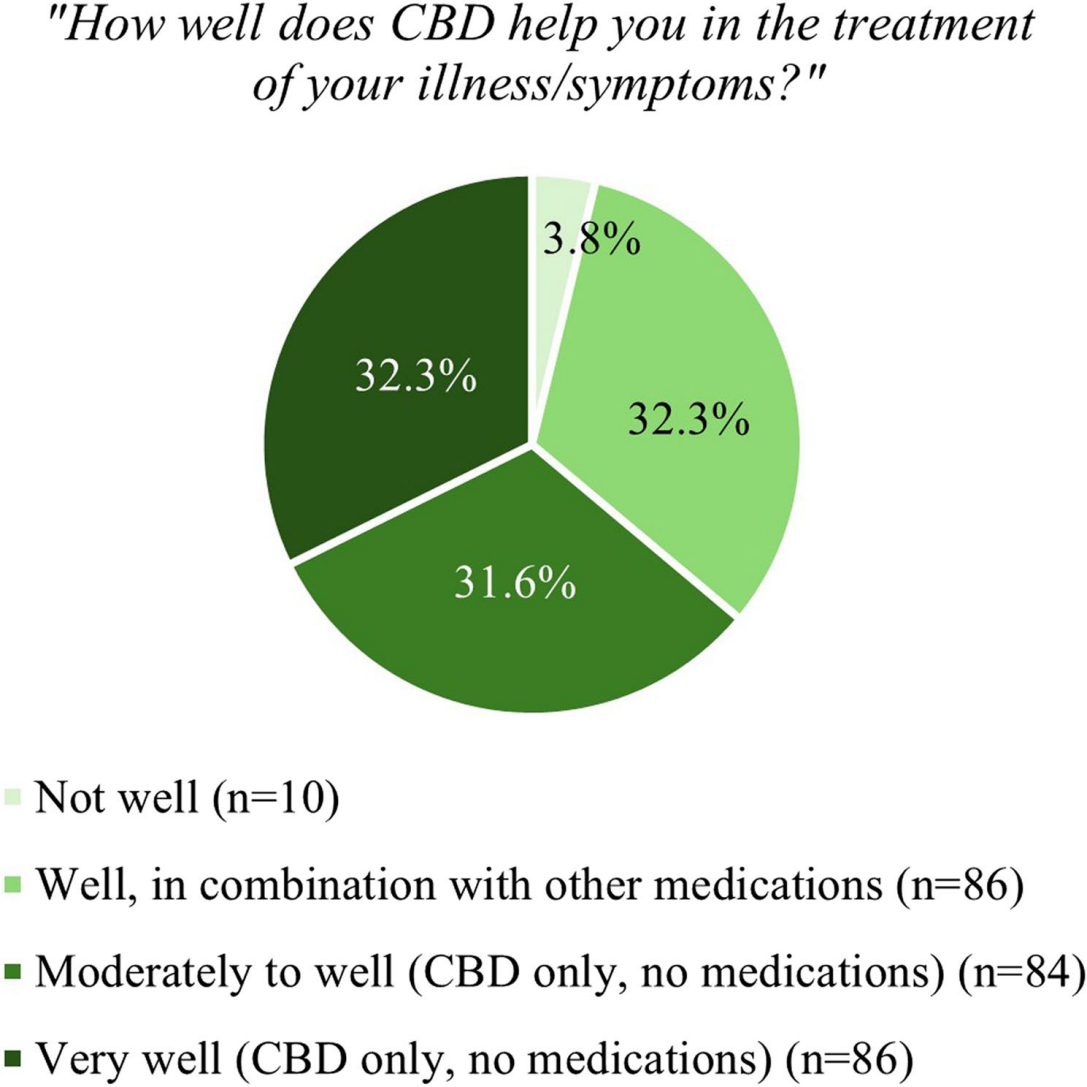
Perceived effectiveness of CBD among users with self-medication as primary motive (single response; *n* = 266)

Among participants who reported self-medication as their primary motive for CBD use (*n* = 266), the perceived effectiveness of CBD varied. A small proportion (3.8%, *n* = 10) indicated that CBD was not effective for the treatment of their illness or symptoms. About one-third of participants (32.3%, *n* = 86) reported that CBD was helpful in combination with other medications. Similarly, 31.6% (*n* = 84) stated that CBD alone was moderately to well effective, while 32.3% (*n* = 86) rated CBD alone as very effective. (Fig. 2) illustrates the perceived effectiveness of CBD among participants using it primarily for self-medication.

The regression model assessing self-medication as the primary motive for CBD use included variables significantly associated with the motive, as indicated by V and p. Predictors in the model were gender, age, occupation, route of administration, frequency, and time of consumption. Education, consumption duration and daily amount were excluded due to non-significant associations. After excluding vocational qualification, no significant multicollinearity was detected among the variables (variance inflation factor [VIF] = 1.88). The overall model was significant (likelihood ratio *x²*(Lachenmeier 2020) = 153.21,*p* <.001), suggesting that at least some of the predictors significantly influenced the dependent variable.

After adjusting for covariates in the logistic model, gender (aOR: 1.50, CI: 0.96–2.33, *p* =.074) was no longer significantly associated with use motive. Age categories revealed that older individuals, had significantly higher odds of using CBD for medical purposes among older individuals, aged 40–49 (aOR: 1.97, CI: 1.05–3.70, *p* =.035), with the highest chance observed for those aged 50 and above (aOR: 2.81, CI: 1.36–5.83, *p* =.005). Occupation was also a significant predictor, with individuals who were not currently employed or retired showed significantly higher odds of using CBD for self-medication (aOR: 3.55, CI: 2.13–5.92, *p* <.001), compared to full-time employees. Regarding the form of consumption, smoking CBD mixed with tobacco and smoking pure CBD were both negatively associated with medical use, compared to sublingual ingestion. Specifically, the odds of using CBD for medical purposes were lower for those smoking CBD mixed with tobacco (aOR: 0.37, CI: 0.22–0.62, *p* <.001) and for those smoking pure CBD (aOR: 0.55, CI: 0.30–0.99, *p* =.046). In terms of frequency of use, CBD users who consumed CBD once a day had significantly higher odds of using CBD for medical reasons compared to those using it at least monthly (aOR: 2.52, CI: 1.23–5.13, *p* =.011). Additionally, individuals who used CBD multiple times a day showed increased odds of using it for medical reasons, though this was not statistically significant (aOR: 1.60, CI: 0.87–2.96, *p* =.132). Furthermore, consumption on an as-needed basis was significantly associated with increased odds of using CBD primarily for self-medication (aOR: 2.72, CI: 1.72–4.29, *p* <.001), as was morning and evening use (aOR: 3.05, CI: 1.42–6.56, *p* =.004), compared to users who reported using CBD only in the evening. Table 3 presents the final model.

**Table 3:** Binary logistic regression predicting self-medication (reference: recreational use) with CBD by sociodemographics and consumption patterns

	aOR ^1)^	95%-CI ^2)^	*p*
Gender			
Male	[Reference]		
Female	1.50	0.96–2.33	0.074
Age groups (years)			
18–24	[Reference]		
25–29	1.65	0.92–2.95	0.095
30–39	1.57	0.89–2.75	0.117
40–49	1.97	1.05–3.70	0.035*
≥ 50	2.81	1.36–5.83	0.005*
Occupation			
Employed full-time	[Reference]		
Employed part-time	1.12	0.64–1.94	0.695
Pupil/student/intern or marginally employed	1.46	0.84–2.56	0.182
Not currently employed or retired	3.55	2.13–5.92	< 0.001*
Frequency of use			
At least monthly	[Reference]		
At least weekly	1.50	0.70–3.22	0.296
Several times per week	1.08	0.56–2.09	0.813
Once a day	2.52	1.23–5.13	0.011*
Several times per day	2.00	0.98–4.08	0.057
Route of administration			
Sublingually	[Reference]		
Smoking (with tobacco)	0.37	0.22–0.62	< 0.001*
Smoking (pure)	0.55	0.30–0.99	0.046*
Vaping	1.18	0.69–2.04	0.549
Other	0.92	0.51–1.68	0.790
Daytime of consumption			
In the evening	[Reference]		
In the morning	1.59	0.57–4.45	0.375
Morning and evening	3.05	1.42–6.56	0.004*
Multiple times per day	1.60	0.87–2.96	0.132
When needed	2.72	1.72–4.29	< 0.001*

* Significant on a threshold of *α* < 0.05

^1)^ Adjusted odds ratio (aOR)

^2)^ Confidence interval (CI)

## Discussion

The present study examined associations between user the primary motive of use, demographic characteristics as well as consumption patterns in a sample of German CBD users. Primary consumption motive was associated with gender, age group, occupation, frequency, route of administration and daytime of consumption. In the adjusted model, consumption patterns, age and occupation remained statistically associated with motive of use; with older users and those who reported being currently unemployed or retired having higher chances of reporting CBD for self-medication. Additionally, the results indicated that daily and as-needed CBD consumption, as well as morning and evening use, were associated with increased odds of self-medication as the primary motive. In contrast, smoking CBD (both mixed with tobacco and pure) was associated with decreased odds of self-medication. Among an adult German sample of regular CBD users, the most frequently reported motives for self-medication were sleeping problems, chronic pain, depression and anxiety. Evidence indicates that cannabis products are often used as a ‘natural’ alternative when conventional treatments have proven ineffective and because they are perceived as preferable to conventional medications, offering natural relief without the intoxicating effects associated with THC-containing products (VanDolah et al. 2019; Wallace et al. 2020). Such expectancies, combined with misleading advertising in the media (Engeli et al. 2025; Moore et al. 2024), may contribute to the use of CBD for self-medication across a wide range of conditions. This may also lead to an overestimation of therapeutic effectiveness, which seems to be reflected in our data, as only a small proportion of participants reported that CBD was not helpful for managing their medical conditions.

### Self-medication with CBD: demographic characteristics and consumption patterns

Consistent with previous findings from the UK and Canada (Goodman et al. 2022; Moltke and Hindocha 2021), women were more likely to consume CBD for self-medication. The gender-related effects may be attributable to the fact that several conditions frequently managed with CBD, as well —such as (premenstrual) pain, anxiety, and depression—are either unique to or more prevalent among women, as previously hypothesized by Goodman and colleagues (Goodman et al. 2022). After adjusting for other predictors, the gender effect was no longer statistically significant. Findings from this sample indicate that consumption patterns, as well as age and occupation, may be a stronger and more reliable predictor than gender. After adjusting for other factors, older age remained positively associated with medical consumption motives, with adults aged 40 and older more likely to use CBD for medical motives, a finding consistent with the results of Geppert et al. (Geppert et al. 2020). Additionally, being unemployed or retired was also associated with primary medical use. These associations may be mediated by a higher burden of disease in older subgroups, indicating that age presents a strong confounder. Primary recreational CBD use was positively associated with administration via smoking (with or without tobacco), while the opposite applied for the consumption for self-medication. The detrimental effects of smoking—especially with tobacco—are associated with various negative health outcomes (U.S. 2024), which might contradict health-conscious decisions. Those findings were consistent with results from Goodmann and colleagues (Goodman et al. 2022), indicating that CBD oil, which is used sublingually is rather used for self-medication, compared to other application forms. Recreational CBD use was mostly consumed in the evening, which indicates that it is predominantly used for relaxation during free. In contrast, medical use was consumed when needed or at a specific time which aligns more closely with conventional medication usage. Additionally, primarily use for self-medication was associated with a higher consumption frequency, which also aligns more closely with conventional medication use.

###  Self-medication with CBD: findings from other countries

Approximately one-third of the present sample used CBD primary for self-medication, while the majority reported using CBD primarily for recreational purposes. These findings are in line with data from France, where treatment of disease only represented the main motivation in one in four cases (Fortin et al. 2021). In contrast, in the U.S., two in three users reported that they used CBD for the treatment of a medical condition (Corroon and Phillips 2018). Evidence suggests country-specific differences in the motive of use and perception of CBD products. Findings indicate that CBD tends to be regarded more as a recreational product in Germany and France, while in the U.S. and the UK it seems to be more frequently used in the context of self-medication. Differences in perception and consumption of CBD products might be driven by national law enforcement policies regarding cannabis. Indeed, comparisons of user motivations and patterns across the EU, U.S., and UK indicate that regulatory frameworks are pivotal in shaping variations in user behaviors (Fortin et al. 2021; Mead 2017). In the U.S., the legalization of cannabis in several states has facilitated greater acceptance of CBD as a medical product and was associated with more favorable health perceptions regarding cannabis consumption (Gali et al. 2021). For instance, the consumption and sale of CBD flowers is more strictly prosecuted in the UK than in Germany or France (Hurt 2023). The dried plants, which is often administered via smoking and associated with recreational use, has high user rates in Germany and France, which potentially underscore the significant influence of legal frameworks on user access and perceptions of CBD products. In conclusion, differences in CBD use motives may exist between countries with varying cannabis and CBD regulations, possibly reflecting differing perceptions of CBD. This is particularly relevant for Germany, where recent cannabis legalization is expected to increase the availability and use of cannabis products, including CBD (Cerdá et al. 2008; Mennis et al. 2023). Future research should examine how regulatory frameworks shape perceptions and consumption motives of CBD, using representative samples to enable valid cross-country comparisons. Surveys should therefore investigate associations between use motives, consumption patterns, and regulatory contexts for medical and recreational cannabis, as well as capture the extent to which cannabis products—particularly cannabidiol—may be advertised in different countries and assess user expectations accordingly.

### CBD for sleep, pain, and depression and anxiety: evidence of effectiveness

The reported medical indications for CBD use align with studies from other countries, which also report anticipated health benefits such as sleep improvement, pain relief, and relief from depression or anxiety (Alayli et al. 2022; Binkowska et al. 2024; Corroon and Phillips 2018; Fortin et al. 2021; Geppert et al. 2020; Goodman et al. 2022; Moltke and Hindocha 2021). There is limited evidence supporting the efficacy of CBD in treating these conditions. A possible explanation for perceived benefits could be the placebo effect, reinforced by non-evidence-based health claims promoted by media and retailers (Moore et al. 2024). Laboratory and animal studies indicate an effect of CBD on the circadian system (Chagas et al. 2013; Ibeas Bih et al. 2015; Leweke et al. 2012; Silvestro et al. 2020; Suraev et al. 2020). Human trials suggest CBD may improve sleep by alleviating symptoms of conditions like pain or anxiety (Notcutt et al. 2004), but its direct impact on the circadian system is still unclear. Placebo effects may largely explain perceived sleep benefits, as a controlled trial by Linares et al. (Linares et al. 2018)showed no acute effect on the sleep-wake cycle. Results from preclinical studies suggest that CBD may alleviate pain by enhancing N-arachidonoylethanolamine signaling, an endocannabinoid involved in pain modulation (Costa et al. 2004; Ibeas Bih et al. 2015). Animal studies have reported a reduction in pain-like behaviors (Soliman et al. 2021). However, reviews on cannabis-based treatments often focus on THC-CBD combinations, complicating the attribution of analgesic effects specifically to CBD alone (Boyaji et al. 2020). In most randomized controlled trials, isolated CBD has shown limited efficacy in pain reduction (Moore et al. 2024). In preclinical studies and animal models, CBD has been shown to interact with the same serotonin receptors that are associated with depression and anxiety (Crippa et al. 2011; Fakhfouri et al. 2019; García-Gutiérrez et al. 2020; Iannotti and Vitale 2021; Ibeas Bih et al. 2015; Moore et al. 2024; Silvestro et al. 2020). This interaction may contribute to its potential therapeutic effects on mood disorders. Although human trials have reported reduced anxiety in patients with anxiety disorders (Bergamaschi et al. 2011; Crippa et al. 2011), recent findings by Gournay et al. (Gournay et al. 2024)suggest that strong user expectations regarding CBD’s anxiety-reducing effects contribute to its perceived effectiveness. Existing studies show heterogeneity in study settings, underscoring the need for research on dose-response, treatment timelines, and administration methods. CBD’s effects vary by dosage, strain, treatment duration, and administration route, with many studies limited by small sample sizes. While molecular properties and animal studies support its therapeutic potential, further clinical investigation is needed to establish efficacy in humans, beyond the placebo effect (Moore et al. 2024). In addition, Given CBD’s potential to interact with other medications (Brown and Winterstein 2019) and induce adverse effects, further investigation into drug-CBD interactions is essential to assess their clinical significance and associated risks.

### Strengths and limitations

This study is the first to identify consumption patterns associated with different use motivations among regular CBD users. The consistency of the findings related to user motivations with previous cross-sectional studies from France, the UK, and the US, strengthens the generalizability of the results. The present study comes along several limitations. First, the study is subject to selection bias due to self-sampling. Individuals with negative experiences regarding their CBD use may be underrepresented, while those more favorable toward CBD products are more likely to participate. This could result in an overestimation of the self-perceived therapeutic effectiveness. Due to the recruitment method of convenience sampling, we do not claim representativeness of the sample. Other limitations involve reliance on retrospective self-reporting, lack of objective measures, and the inability to establish causality due to the cross-sectional design. Reliance on non-validated questionnaires is a limitation, highlighting the need for CBD-specific instruments, as THC-related questionnaires may not adequately capture CBD-specific outcomes. At present, only the *Cannabidiol Outcome Expectancies Questionnaire (CBD-OEQ)*is validated (Walukevich-Dienst et al. 2022), but it measures psychometric properties, such as expectancies and endorsement, rather than motives or consumption patterns. With regard to validated questionnaires, future studies should include additional confounders, as the regression model explained only around 16% of the variance. Relevant factors include comorbidities and physician-diagnosed chronic conditions, rather than relying solely on self-reported medical indications, which do not constitute a medical diagnosis. Moreover, the use of other substances such as THC or tobacco should be assessed, as they may constitute important confounders. In addition, the response option*‘addiction cessation’* in the predefined list of medical indications should be specified more precisely to enable a more accurate investigation of associations between CBD use and other substances. These methodological shortcomings of the survey instrument highlight the need for validated CBD-specific outcome measures. In addition, the lack of distinction between CBD and THC users may have confounded the observed therapeutic effects.

## Conclusion

This study represents the first in depth-analysis of sociodemographic characteristics and consumption patterns among individuals who primarily use CBD for self-medication versus recreational purposes. Distinct consumption patterns were observed, aligning with either recreational use or self-medication motives. The findings provide preliminary insights into potential associations between legal frameworks and consumption behaviors across countries with differing regulations. The results underscore the importance of developing standardized regulatory policies for various CBD products. Despite molecular evidence and results from animal models, further clinical trials are needed to establish the true efficacy of CBD. Future research should focus on examining dose-response relationships, different routes of administration, effects of isolated CBD, and assessing potential CBD-drug interactions. Additionally, RCTs are essential to differentiate the true therapeutic effectiveness from placebo effects, which are often reinforced by media or false advertisement.

## Data Availability

No datasets were generated or analysed during the current study.
